# Gene Characterization Index: Assessing the Depth of Gene Annotation

**DOI:** 10.1371/journal.pone.0001440

**Published:** 2008-01-23

**Authors:** Danielle Kemmer, Raf M. Podowski, Dimas Yusuf, Jochen Brumm, Warren Cheung, Claes Wahlestedt, Boris Lenhard, Wyeth W. Wasserman

**Affiliations:** 1 Center for Genomics and Bioinformatics, Karolinska Institute, Stockholm, Sweden; 2 Department of Biochemistry and Molecular Biology, University of British Columbia, Vancouver, Canada; 3 Centre for Molecular Medicine and Therapeutics, Child and Family Research Institute, Department of Medical Genetics, University of British Columbia, Vancouver, Canada; 4 Department of Statistics, University of British Columbia, Vancouver, Canada; 5 Molecular and Integrative Neurosciences Department, The Scripps Research Institute, Jupiter, Florida, United States of America; 6 Computational Biology Unit, Bergen Center for Computational Science, Sars International Centre for Marine Molecular Biology, Unifob AS, University of Bergen, Bergen, Norway; Centre de Regulació Genòmica, Spain

## Abstract

**Background:**

We introduce the Gene Characterization Index, a bioinformatics method for scoring the extent to which a protein-encoding gene is functionally described. Inherently a reflection of human perception, the Gene Characterization Index is applied for assessing the characterization status of individual genes, thus serving the advancement of both genome annotation and applied genomics research by rapid and unbiased identification of groups of uncharacterized genes for diverse applications such as directed functional studies and delineation of novel drug targets.

**Methodology/Principal Findings:**

The scoring procedure is based on a global survey of researchers, who assigned characterization scores from 1 (poor) to 10 (extensive) for a sample of genes based on major online resources. By evaluating the survey as training data, we developed a bioinformatics procedure to assign gene characterization scores to all genes in the human genome. We analyzed snapshots of functional genome annotation over a period of 6 years to assess temporal changes reflected by the increase of the average Gene Characterization Index. Applying the Gene Characterization Index to genes within pharmaceutically relevant classes, we confirmed known drug targets as high-scoring genes and revealed potentially interesting novel targets with low characterization indexes. Removing known drug targets and genes linked to sequence-related patent filings from the entirety of indexed genes, we identified sets of low-scoring genes particularly suited for further experimental investigation.

**Conclusions/Significance:**

The Gene Characterization Index is intended to serve as a tool to the scientific community and granting agencies for focusing resources and efforts on unexplored areas of the genome. The Gene Characterization Index is available from http://cisreg.ca/gci/.

## Introduction

Elucidation of the function(s) for each human protein-encoding gene has been a prominent challenge in biomedical research after the completed deciphering of the sequence of the human genome. Systematic characterization projects have been launched, ranging from the ENCODE project for detailed genome annotation [1] to the phenome projects to identify phenotypes generated by mutations of human gene orthologs in model organisms [2]–[4]. These efforts were undertaken, in part, to evoke new insights into the functions of uncharacterized genes revealed through the successful sequencing of the human genome. At the level of basic human curiosity, scientists are drawn to these uncharacterized genes, for it is the deciphering of the functions of these genes which offers the greatest potential to gain fundamental insights into novel biological processes; to peer into the unknown. The therapeutic and financial benefits associated with successful identification of genes that are suitable targets for pharmaceutical research and informative biomarkers for treatment selection stands as another strong motivator.

The arsenal of the modern molecular researcher, when directed at specific genes, can elucidate properties that offer glimpses of underlying functions. In the laboratory we can determine specific phenotypic effects of a gene when disrupted in model organisms, where the encoded protein localizes within the cell, the spatio-temporal coordinates of gene activity, the function in cells or model organisms through biological assays, and further techniques *ad infinitum*. To unleash these often expensive and time-consuming studies, researchers (and funding agencies) are usually motivated by preliminary glimmers of functional knowledge. However, with the goal of comprehensiveness, attempts to explore genome function in an unbiased manner have been made. In the ENCODE project, undertaken by a portion of the global research community to systematically annotate functions for 1% of the human genome, a portion of the genome was selected for study for the glaring absence of knowledge about the genes in the region [1]. The Allan Brain Atlas [5] places a premium on the systematic study of expression in the mouse brain of uncharacterized genes. In the pharmaceutical industries, gaining insights into the functions of uncharacterized genes can offer a direct and meaningful path to successful drug target identification.

However, based on available methodologies, it has been challenging to divide genes into classes depending on functional knowledge and to extract sets of uncharacterized genes from the genome. The characterization state of each gene exists in the eye of the beholder; each scientist brings a distinct perspective to the interpretation of the characterization status of a gene. While scientists can select individual genes, judged as being scarcely annotated based on available information in multiple data repositories, no quantitative measurement of annotation status exists widely applicable to sets of genes of particular interest to further experimental study. However, previous studies have shown that human opinion, based on specific sets of predictors, can be quantified and predicted. In fact, researchers have surveyed the perception of human beauty and developed computational methods capable of accurately predicting consensus opinions by collecting human ratings and developing machine learning procedures based on those ratings [6], [7].

With the goal of establishing a quantitative and universal system for measuring the annotation status of human genes, we developed the Gene Characterization Index (GCI), a bioinformatics procedure to quantitatively assign a characterization score to each human gene relying on collected opinions from the global research community. In this report, based on training data derived from a reference collection of genes with assigned characterization scores from over 50 scientists worldwide, we constructed a classification function that successfully predicts the characterization of human protein-encoding genes. By applying the method to well-studied classes of genes followed by comparison to the depth of annotation in the Gene Ontology (GO) system [8], we confirmed the accurate prediction of the level of functional characterization by the GCI. At the genome scale, we integrated GCI values across all human protein-encoding genes to determine the characterization status of the human genome. We found that the progress made by the research community to assign functions to human genes after the release of the first draft of the human genome in 2000 was well-reflected by an increase of the average GCI score across the genome over time.

At a finer grain, analyzing classes of pharmaceutically relevant gene families such as G protein-coupled receptors, nuclear receptors and ion channels, we revealed specific characteristics of the different groups and highlighted opportunities for the identification of hitherto overlooked novel drug targets within those therapeutically relevant protein families with potentially important roles in the treatment of various diseases.

The GCI is the first automated method for quantitatively assessing the extent to which each gene is annotated. By applying the GCI scoring system on a genome scale, we identified a large portion of the human genome, likely including groups of genes with potentially interesting applications, essentially neglected by the scientific community. By drawing attention to these groups of weakly annotated genes, the GCI could help advance genome characterization in an unbiased manner. Translated to the single gene level, our scoring system is intended to help direct funding agencies to these neglected areas and to guide the level of analysis that should be funded. It serves as a resource for focusing experimental efforts and can provide both computational and laboratory scientists with opportunities to demonstrate the novelty of current findings and the utility of new methods.

## Results

### Implementation of the Gene Characterization Index

To create a quantitative method for assigning characterization scores to each human gene, a representative reference collection of scores for a subset of genes was required. This collection served both as training data for determining predictive characteristics for a perceived characterization state, as well as test data for determining the reliability of the predictive methods generated. To create a broadly representative method, the reference collection of gene characterization scores was created through a global survey of life sciences researchers with diverse scientific backgrounds. We asked 52 scientists worldwide to assess a sample of genes and assign a score within a 10-point scale, with 1 indicating a completely uncharacterized gene and 10 a gene that is fully described. As the GCI directly reflects the scores obtained in the survey, the survey is described in detail.

### Reference gene definition and annotation source

Both for the survey and the ultimate production of the GCI, it was necessary to define a reference set of human genes. Such lists could be obtained from a variety of sources, each with unique characteristics. In addition to a list of genes, we desired a system that provided diverse functional annotations, as these gene characteristics constituted the variables that could be evaluated and quantitated by a scoring function. We selected the Entrez Gene database [9] as an appropriate data source, as it met the above-mentioned requirements and was expected to maintain data quality with regular updates. To retroactively measure the progress of human genome annotation, we further required a gene annotation source providing access to releases over a multi-year period. For this purpose, we selected the GeneLynx database [10], as it was the most accessible system with resources comparable to Entrez Gene for which annotations could be obtained from an extended time period. Finally, we selected the set of “training” genes from release 1.2 of the GeneLynx database (June 2003) in a 2-stage process to insure a balanced representation of genes with diverse characteristics. We randomly chose an initial set of 90 genes from the subset of genes for which cDNA sequences were available in GenBank [11]. In addition, to insure the inclusion of genes with minimal functional characterization, we included 10 genes represented only by expressed sequence tags (ESTs).

### Evaluator ratings

To gather scores for the reference genes we developed a web-based survey system. Each participating scientist was assigned 10 genes (including 1 EST-only gene) and asked to provide a characterization value. An optional guide to scoring was made available to the evaluators to assist in determining scores (see online Supplementary Material). Each gene was assessed by multiple biologists to allow the determination of an average score. We sought at least 3 independent scores for each gene in the reference collection. As genes were randomly assigned, the actual number of responses per gene varied, with a minimum of 3 and an average of 4.6. Supplementary Table S1 describes the complete list of reference genes that was used in the survey together with average evaluator ratings for each gene. Supplementary Figure S1 shows the distribution of evaluator ratings.

### GCI classifier selection

Once we had collected evaluator ratings for the reference genes, we set out to train a predictor from those ratings through the application of machine learning procedures including linear models (LM), regression trees (RT), neural nets (NN), support vector machines (SVM) [12] and multivariate additive regression splines (MARS) [13]. For the implementation of universal predictors of gene annotation status, we first selected sets of gene characteristics used as classifiers from the training data. As indicated previously, we selected historical releases of the GeneLynx database [10] as the source for annotation over an extended time period. Similar to the more recent Entrez Gene [9], GeneLynx provided links to a diverse array of online resources with gene-specific information; these links were automatically compiled from numerous systems. We treated the compilation of resources for each gene as gene characteristics that could be quantified. As those resources have changed over time, we restricted the GCI training data to those resources that had been recorded in GeneLynx since 2001. In addition to these core GeneLynx annotations, we also considered gene-related articles published prior to the survey; the article counts were based on references in the Entrez Gene and SwissProt databases.

Initially, we compiled a total of 40 gene characteristics for the reference genes (see Supplementary Table S2). We then created each predictor vector by determining the total number of unique attributes for each gene, followed by post-processing of each field, including z-score normalization (see online Supplementary Material). As a consequence, the list of 40 attributes was subsequently reduced to 16 by removal of gene characteristics not represented in all GeneLynx releases, and further by selection of a single representative from highly correlated groups (Table 1 and 2). We considered attributes with a correlation of 0.7 or higher similar, thus becoming candidates for removal.

**Table 1:** Classification model attributes used by the MARS model.

Attribute	Description
GBACC	GenBank sequences
INTERPRO	Interpro domains
KEGG	KEGG pathways
MEDLINE	MEDLINE references in Entrez Gene
OMIM	OMIM references
SPID	SwissProt protein links

**Table 2:** Additional available classification model attributes.

Attribute	Description
DBSNP	Single nucleotide polymorphisms
ENSEMBL	EnsEMBL transcripts
GO	Gene Ontology terms
HOMOLOGENE	Non-human homologous sequences
NAME	Gene symbols and alias symbols
PDB	PDB protein structures
PRINTS	PRINTS protein fingerprints
PROSITE	Prosite references
REFSEQ	RefSeq sequences
TREMBL	TrEMBL links

Available attributes for use in the statistical model for defining gene annotation state include single nucleotide polymorphisms (SNPs) describing DNA sequence variations often linked to disease, genomic sequences derived from GenBank [11], InterPro [14], PRINTS [15] and PROSITE [16] domains assigning genes to families, Gene Ontology (GO) annotations [8] using consistent descriptions for gene function, reports in KEGG [17] integrating genes into cellular pathways, associations in OMIM linking genes to known diseases with a genetic component (http://www.ncbi.nlm.nih.gov/omim/), RefSeq accessions [18] pointing to a gene's curated annotation level, SwissProt entries [19] providing manually curated information on a gene's protein product, PubMed (http://www.pubmed.gov) literature references, PDB [20] protein structure information, HomoloGene (http://www.ncbi.nlm.nih.gov/HomoloGene/) linking genes to homologous sequences in other species and descriptive annotations such as gene name, symbol and functional description.

### Model selection

Based on the performance observed and the clarity of the underlying procedure, we selected a MARS model for the final function. It is important to note that the selection of the statistical approach had limited importance, as several methods performed comparably well in the model validation step. The ultimate model produced scores based on 6 attributes (Table 1). We explored models using less attributes, but found that 6 attributes performed best. Full documentation of the performance review and validation can be found in the online Supplementary Material.

**Figure 1:**
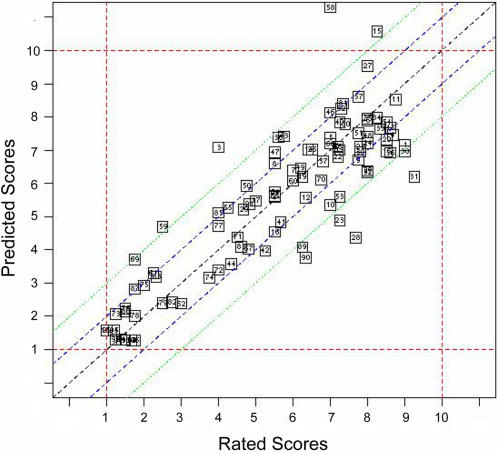
GCI Model Cross-validation Performance. GCI Predictor Performance–Leave-One-Out cross-validation results for the final GCI predictor model utilizing the MARS method on z-score normalized data. The X-axis displays average evaluator assigned scores, while the Y-axis displays the predicted scores for each gene in the leave-one-out cross validation analysis (the score assigned when the gene was not included in the training data). As observed, the MARS model can assign scores greater than 10 (in all further analysis such scores are rounded down to 10).

The MARS method assigned scores within +/−1 of the average reviewer score for 57% of reference genes and 81% of assigned scores were within +/−2 of the reviewer assigned scores (Figure 1). However, for a few genes that scored greater than +/−2 from the mean, we conducted a manual review to assess the overall annotation levels by enumerating available gene characteristics possibly explaining the discrepancy between evaluator ratings and computational predictions. For example, SOCS box-containing WD protein SWiP-1 (*WSB1*) (Entrez Gene ID 26118), rated at 2.5 but was predicted at 5.3. This gene presented a moderate number of associated publications in PubMed, contained mainly protein domain-specific annotations and therefore, was scored low by human evaluators. However, inclusion of *WSB1* in the KEGG database of cellular pathways, the OMIM database of disease associations, the SwissProt protein database, as well as the presence of a number of SNPs suggested a curated level of functional annotation. As a consequence, we judged that the predicted score more accurately reflected the functional understanding of the gene.

### Performance of the Gene Characterization Index

To assess the capacity of the GCI scoring model to successfully predict gene annotation levels, we integrated characterization scores across all predicted human genes and further compared those scores to information content from the Gene Ontology (GO) gene annotation system [8].

### Genome-scale scoring and temporal annotation changes

Since the release of the first draft of the human genome sequence in 2000, concerted efforts of the scientific community have resulted in the successful delineation and functional annotation of a large body of predicted human genes. We reasoned that, following genome evolution over the years since its release, we could follow and quantify the expansion of functional knowledge via an increase in the average GCI score over time. To compare successive versions of the human genome, we applied the final GCI scoring model to all genes present in either the Entrez Gene database [9] for recent genome releases, or in the GeneLynx database [10] for historical genome versions.

**Figure 2:**
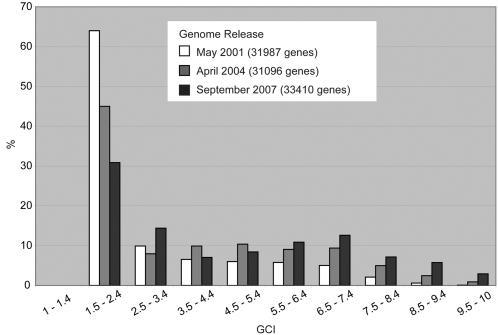
Genome-wide GCI Score Distribution. Histogram displaying the frequency of scores observed in the analysis of genes at 3 different time points after the release of the first draft of the human genome sequence. Genes based only on predictions and/or EST sequences have been removed (∼3000 genes in 2007 data).

The histogram in Figure 2 shows the distribution of GCI scores across all human genes taken at 3 different time points. In the earliest version, 64% of all genes (20475 of 31987 genes) clustered at the bottom of the scale with scores lower than 2.5, and less than 3% (885 genes) scored at 7.5 or higher, reflecting the scarce overall annotation level of the human genome. Successive genome releases showed an important decrease in the low-scoring group of genes with 30.9% of scores (10308 of 33410 genes) under 2.5 for the latest release in September 2007. Also apparent from the histogram was the steady increase in high-scoring genes over time. The portion of genes scoring above 7.5 rose from merely 2.8% (885 of 31987 genes) in May 2001 to 8.3% (2588 of 31096 genes) in April 2004 reaching 15.8% (5286 of 33410 genes) in the latest release. Overall gene numbers fluctuated between successive genome releases due to changing genome annotations and transcript-to-gene mappings. To provide some perspective to the reader, we assembled a subset of the highest and lowest scoring genes from the September 2007 release presented in Tables 3 and 4.

**Table 3:** Sets of genes with extreme characterization scores: sampling of well-characterized genes.

Gene Name	Symbol	GCI	Gene ID	Description
Lamin A/C	LMNA	10.0	4000	Lamin-A/C (70 kDa lamin) (Renal carcinoma antigen NY-REN-32)
Collagen, type II, alpha 1	COL2A1	10.0	1280	Collagen, type II, alpha 1 (primary osteoarthritis, spondyloepiphyseal dysplasia, congenital)
Phosphatase and tensin homolog	PTEN	10.0	5728	Phosphatase and tensin homolog (mutated in multiple advanced cancers)
Tumor protein p53	TP53	10.0	7157	Cellular tumor antigen p53 (Tumor suppressor p53) (Phosphoprotein p53) (Antigen NY-CO-13)
Fibroblast growth factor receptor 2	FGFR2	10.0	2263	Fibroblast growth factor receptor 2 (bacteria-expressed kinase, keratinocyte growth factor receptor, craniofacial dysostosis 1, Crouzon syndrome, Pfeiffer syndrome, Jackson-Weiss syndrome)
Titin	TTN	10.0	7273	Titin (EC 2.7.11.1) (Connectin) (Rhabdomyosarcoma antigen MU-RMS- 40.14)
Peroxisome proliferator-activated receptor gamma	PPARG	10.0	5468	Peroxisome proliferator-activated receptor gamma (PPAR-gamma)
Paired box 6	PAX6	10.0	5080	Paired box protein Pax-6 (Oculorhombin) (Aniridia type II protein)
Melanocortin 1 receptor	MC1R	10.0	4157	Melanocortin 1 receptor (alpha melanocyte stimulating hormone receptor)
V-Ki-ras2 Kirsten rat sarcoma viral oncogene homolog	KRAS	10.0	3845	GTPase KRas precursor (K-Ras 2) (Ki-Ras) (c-K-ras) (c-Ki-ras)

**Table 4:** Sets of genes with extreme characterization scores: sampling of scarcely characterized genes.

Gene Name	Symbol	GCI	Gene ID	Description
KIAA1833-like		1.5	377711	LOC377711 KIAA1833-like
LOC730919		1.5	730919	Hypothetical protein LOC730919
Family with sequence similarity 24, member A	FAM24A	1.5	118670	FAM24A family with sequence similarity 24, member A
Chromosome 1 open reading frame 192	C1orf192	1.5	257177	C1orf192 chromosome 1 open reading frame 192
LOC284428		1.5	284428	LOC284428 similar to methyl-CpG binding domain protein 3-like 2
LOC388910		1.5	388910	RP3-474I12.5 hypothetical LOC388910
Family with sequence similarity 90, member A3	FAM90A3	1.5	389611	FAM90A3 family with sequence similarity 90, member A3
LOC400723		1.5	400723	Hypothetical LOC400723
LOC400856		1.5	400856	Hypothetical gene supported by AK123815
LOC440776		1.5	440776	Hypothetical LOC440776

### Qualitative adaptation of scoring model

To qualitatively assess the increase in gene characterization over time, we analyzed contributions of different annotation sources assembled in Entrez Gene and GeneLynx to rising gene annotation levels. We counted entries for each attribute selected by the MARS model. Analyzing annotation changes between July 2006 and September 2007, it became apparent that the increase in the number of PubMed references largely dominated all other attributes, and thus, publications could be considered the most important contributor to the recent advancement of gene annotation levels (Table 5). In addition to publication in peer-reviewed journals, recent expansion of the KEGG database of cellular pathways contributed moderately to improved annotation. While new disease associations recorded in the OMIM database and new SwissProt entries contributed to increased annotation levels, there was a decrease in the number of protein domain family associations reported in InterPro likely reflecting a change in genome annotation procedures.

**Table 5:** Individual contribution of attributes to rising annotation levels.

Attributes	July 2006	Sept 2007	Change (%)
MEDLINE	229728	338417	+47.3
GBACC	190077	227822	+19.9
KEGG	8444	9618	+13.9
OMIM	14135	15586	+10.3
SPID	14306	15631	+9.3
INTERPRO	51087	44759	−12.4

(GBACC = GenBank accession, SPID = SwissProt identifier).

After investigating characteristics of the more recent genome annotation, we analyzed earlier developments directly following the release of the first genome sequence drafts. Historical annotation changes were reflected by changing attributes selected by the MARS model in the GeneLynx database. While there had been a steady increase in the number of PubMed references across all time points, earlier releases were essentially marked by an increased association of cDNAs-to-genes and the designation of official gene names and symbols. Other characteristics of functional gene annotation were the inclusion of SNPs, increases in Gene Ontology annotations, InterPro domains, KEGG pathways, and OMIM disease associations.

### Comparison to Gene Ontology annotation levels

The Gene Ontology (GO) project is an effort to standardize gene descriptions using a predefined vocabulary of functional terms [8], and GO terms are now widely used to functionally annotate genes and their protein products [21]. GO is a highly curated system that uses 3 structured ontologies to describe genes in terms of their associated *molecular function*, *biological process* and *cellular component*. As gene annotation levels deepen, the hierarchy of specialized GO terms extends, describing the gene in greater detail. Functional comparison of sequences annotated with GO terms can be performed based on semantic similarity measures like the ones developed by Resnik [22].

**Figure 3:**
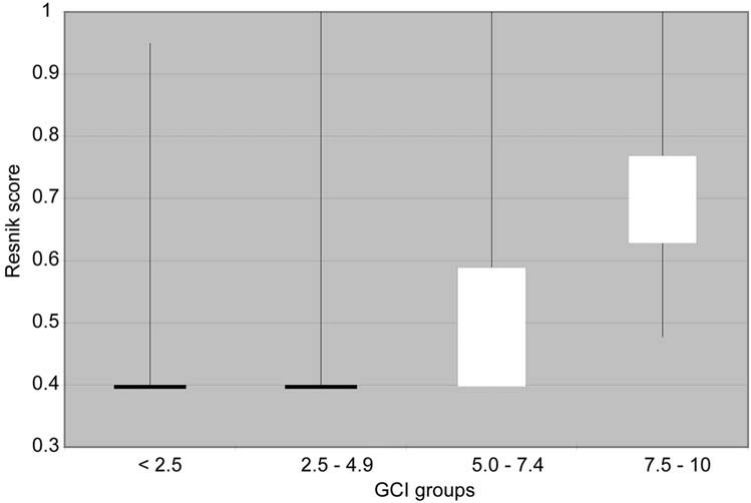
Resnik Scores for Depth of GO Gene Annotation Correspond with GCI Scores. The Resnik score describes the granularity of annotations attached to each gene. There is an overall Pearson correlation of 0.6 between GCI and Resnik scores. The distribution plot shows the distribution of Resnik scores for ranges of GCI scores.

For genes drawn from the latest genome release (September 2007) with GO annotations, we extracted the maximum information content for each gene by using Resnik's similarity measure reflecting the granularity of the GO terms assigned to a given gene. We then compared these raw information content scores to the GCI scores obtained with our scoring model using Pearson correlations. For the comparisons, we selected equal numbers of genes from each bin of GCI scores (e.g. 1–1.4, 1.5–2.4, etc.; see Figure 2 for bins) and calculated overall correlations. Comparing all GCI scores to the GO *molecular function* category using Resnik scores, we observed a Pearson correlation coefficient of 0.59. We also combined bins into larger groups representing increasing annotation levels (low <2.5, medium low 2.5–4.9, medium high 5.0–7.4 and high 7.5–10.0) and examined differences between GCI and GO (Figure 3). Genes with higher scores agreed best with the Resnik scores for GO *molecular functions*, while genes with low scores showed weaker correspondence. No strong correlations were observed for GO *biological process* and *cellular compartment* categories (data not shown). The positive correlation between Resnik scores of GO *molecular function* and GCI scores further validated our automated scoring model as an accurate reflection of the depth of gene annotation levels.

### Application of the Gene Characterization Index

#### Analysis of drug target gene families

Using the predictions of functional gene annotation levels from the GCI scoring system, we investigated the properties of annotation for a variety of gene sets relevant in medicine and drug development. Over the past few decades, 3 gene families have stood out as the most common drug targets: G protein-coupled receptors (GPCRs), nuclear receptors (NRs), and ion channel proteins (ICs) [23]. We extracted all gene members of these families from appropriate resources and, using DrugBank as a resource for drug data and protein target information [24], divided them into known drug targets and non-drug targets. By analyzing each family for its annotation status, we uncovered family-specific characteristics and revealed potential opportunities to study neglected members of these biologically relevant gene classes, as well as to discover new drug targets.

#### G protein-coupled receptors

**Figure 4:**
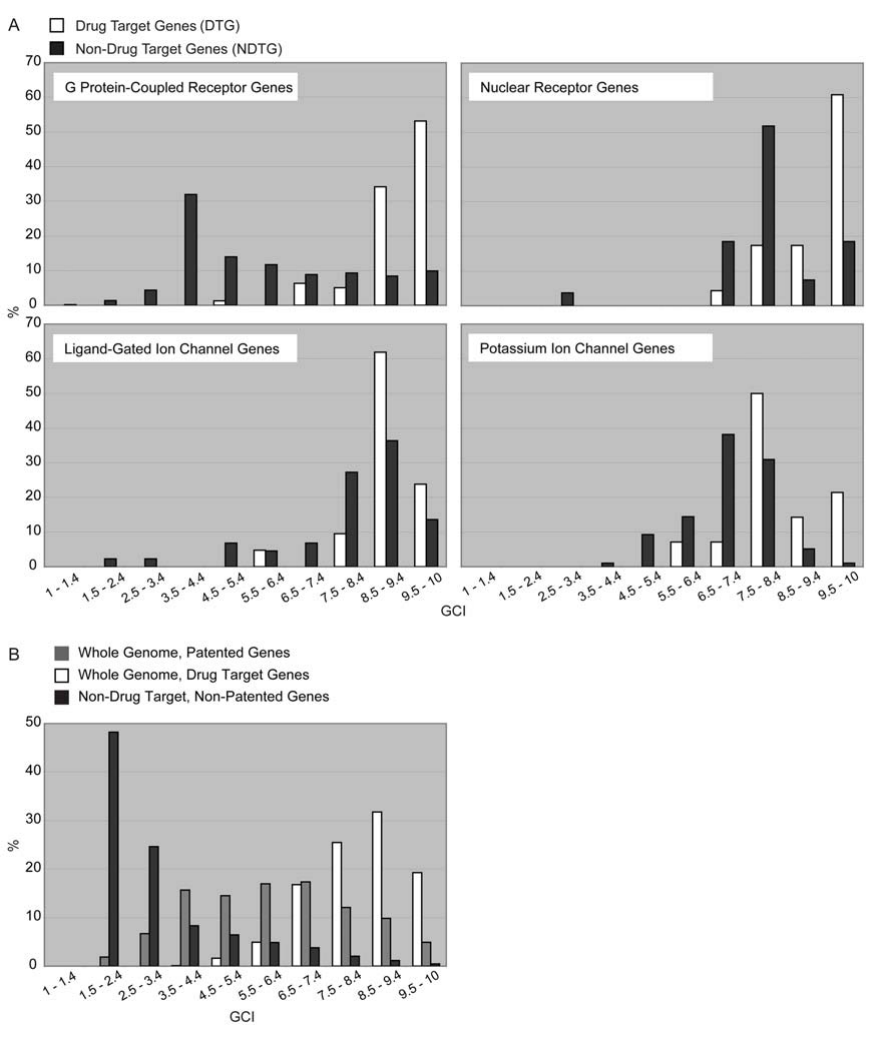
A. Distribution of GCI Scores for Genes in Selected Protein Families and Classes. 750 G Protein-Coupled Recptors: 79 DTG, 671 NDTG; 50 Nuclear Receptors: 23 DTG, 27 NDTG; 66 Ligand-Gated Ion Channels: 21 DTG, 45 NDTG; 111 Potassium Ion Channels: 14 DTG, 97 NDTG. B. Genome-wide GCI Score Distribution for Drug Targets, Patented and All Other Genes. Based on genome release July 2006: 1095 drug targets, 14237 patented, 14913 non-target, non-patented genes. 10867 non-targeted, non-patented genes were highly uncharacterized with GCI scores <3.5.

GPCRs, targeted by nearly one third of currently marketed drugs [23], are diverse in structure and function, and, with their broad evolutionary conservation, are considered the oldest cellular machineries devoted to signal transduction. From the G Protein-Coupled Receptor Data Base (http://www.gpcr.org/) [25] and GO annotations, we extracted a set of 750 GPCRs for further analysis. Applying annotations from DrugBank, we divided the set into GPCRs of FDA-approved drug targets (79) and receptors previously not targeted by pharmaceuticals (671) and applied the GCI scoring system. As apparent from Figure 4A, over 80% of drug target GPCRs presented deep annotation levels and scored between 8.5 and 10.0. This group included highly relevant drug targets such as the angiotensin receptor (*AGTR1*), serotonin receptor (*HTR2A*), and endothelin receptor (*EDNRB*). However, analyzing GPCRs not targeted by small molecule drugs revealed that the majority had remained weakly characterized with GCI scores for over 60% of genes ranging from 1.5 to 5.4 highlighting opportunity for further study and development of new targets within this highly relevant gene family. For example, G protein-coupled receptor 137C (*GPR137C*, Entrez Gene ID 283554, GCI = 3.23), a weakly annotated GPCR, is linked to a single PubMed publication associating the gene's transcription to a gastric cancer gene expression profile [26]. Another example is the 7 transmembrane helix receptor *LOC440683* (Entrez Gene ID 440683) scoring at 2.16 with reported rhodopsin-like receptor activity and no further functional information associated. It should be noted, however, that the group of low-scoring GPCRs could be partly accounted for by the inclusion of olfactory receptors, a subclass with limited therapeutic potential. Comparing the non-drug target group of GPCRs with the GenBank patented sequence repository showed that 537 genes (80%) had patents associated with them.

#### Nuclear receptors

Based on GO annotations and the Nuclear Receptor Database (http://www.receptors.org/NR/) [27], we extracted 50 NRs for further analysis. This second largest group of current drug targets functions as ligand-activated transcription factors and regulate core cellular processes such as cell growth and differentiation, inflammatory responses and metabolism. Their important role in physiology and the ability to regulate their functional activity with synthetic small molecules has rendered this gene family a favorite target for drug discovery [28]. Subjecting NRs to the same analysis as GPCRs, we found that 22 of 23 drug target NRs presented GCI scores of 7.5 and above (Figure 4A). Similarly, the distribution of GCI scores for all 27 non-targeted NRs was high (average GCI = 7.8) reflecting the intense scrutiny given to these proteins. Across all NRs, a single gene obtained a low GCI score at 2.9. This gene (Entrez Gene ID 55566), coding for the estrogen receptor-like p65 protein and linked to colorectal cancer through 2 PubMed publications, lacked additional functional annotation making it a particularly interesting candidate for further study. As shown for GPCRs, the majority of the non-drug target NRs (24) had patents associated with them.

#### Ion channels

ICs constitute the third major class of current drug targets including ligand- and voltage-gated ion channels. The family of ligand-gated ion channels (LGICs) comprises several superfamilies and their physiological activity controls information flow in the brain, thus becoming a relevant class of targets for drugs treating disorders such as epilepsy and anxiety [29]. From the Ligand-Gated Ion Channel database (http://www.ebi.ac.uk/compneur-srv/LGICdb/LGICdb.php) [30] we extracted 66 genes, of which 21 coded for FDA-approved drug targets. Comparable to NRs, 86% of drug target LGICs scored above 7.5, whereas the distribution of GCI scores for non-drug target LGICs was more widely distributed with 38% of genes scoring below 7.5 (Figure 4A). Several low-scoring, non-drug target LGICs coded for weakly annotated subunits of well-known receptor complexes: the alpha 4 subunit of the glycine receptor (*GLRA4*, Entrez Gene ID 441509, GCI = 1.5), the rho3 subunit of the gamma-aminobutyric acid (GABA) receptor (*GABRR3*, Entrez Gene ID 200959, GCI = 3.3), and several subunits of the type 3 receptor for 5-hydroxytryptamine (serotonin). It should be noted that the pool of non-drug target LGICs may include subunits of receptor complexes already targeted by small molecule compounds, as illustrated by the GABA_A_ receptor gamma 2 subunit (*GABRG2*), with a GCI score of 10.0 and no current records in DrugBank. The gene's mutated isoforms have been implicated in epilepsy [31] and, as a subunit of the type A GABA receptor, this gene is part of a protein complex heavily targeted by pharmaceuticals. However, weakly annotated subunits of already targeted protein complexes may present opportunities for the development of novel drugs targeting new sets of targets within protein complexes with known therapeutic potential. As for GPCRs and NRs, the large majority of non-drug target genes (89%) were associated with patents.

Another important class of ICs, Potassium Ion Channels (KICs), associated with action potentials and intercellular signaling, perform a wide variety of functions in both excitable and non-excitable cells and thus, have been recognized as potential drug targets [32]. From the KChannelDB (http://www.receptors.org/KCN/), we extracted 111 KIC genes, 14 of which were targeted by small molecule drugs according to DrugBank. Applying the GCI scoring system to the drug targets, we observed that 9 of 14 KICs obtained scores at 7.5 and above, and that no gene scored below 5.5 (Figure 4A). The analysis of the non-drug target KICs yielded a somewhat different distribution with almost half of the genes (47%) scoring at medium high levels (5.5–7.4), and an important portion (22%) in the range of moderate to weak annotation levels (<5.5). Several of these moderately annotated genes were members of a group of potassium channels containing tetramerisation domains. Non-targeted KICs, and thus interesting for further study, included the potassium channel subfamily T member 1 (*KCNT1*, Entrez Gene ID 57582, GCI = 4.8) and member 2 (*KCNT2*, Entrez Gene ID 343450, GCI = 5.0), the potassium channel subfamily K member 18 (*KCNK18*, Entrez Gene ID 338567, GCI = 4.2), the potassium voltage-gated channel shaker-related subfamily member 7 (*KCNA7*, Entrez Gene ID 3743, GCI = 5.7), and the KCNE1-like membrane protein (*KCNE1L*, Entrez Gene ID 23630, GCI = 6.4). Similar to the rates for other drug target gene families examined, we observed that 88 of 97 (91%) non-drug target KICs were associated to patents in GenBank.

#### Genome-wide analysis of drug target and patented genes

To expand the analysis of GCI distributions to the whole genome, we extracted the entire set of drug targets from DrugBank (1095 proteins) and all patented genes reported in NCBI's patent nucleotide sequence database (14295 genes). Figure 4B illustrates the genome-wide distribution of GCI scores for the different gene classes. Similar to the distribution pattern observed for specific drug target gene families, drug target genes in the genome clustered at the high end of the scoring scale with over 75% having GCI scores above 7.5. Patented genes were scattered across the range of scores with a smaller fractions at the low end (i.e., 1.9% receiving scores <2.4). Removing all drug target and patented genes from the genome, we applied the GCI scoring model to the remaining 14913 genes. Tellingly, over 70% of the non-patented non-target genes were highly unexplored with GCI scores <3.5. The remainder of genes was distributed across the entire scale with decreasing numbers for increasing scores.

#### Effect of patent filing on gene characterization

**Figure 5:**
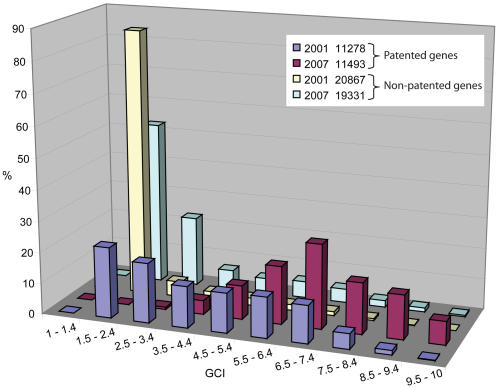
Evolution of Patented versus Non-Patented Genes between 2001 and 2007. Histogram presenting substantial differences in annotation progress between patented and non-patented genes. Fluctuating gene numbers due to changes in genome annotations and transcript mappings.

Since applications for patents on DNA sequences became common practice in the early 1990's [33], critics have raised a number of objections against the patenting of biological material. The debate has remained vivid to the current day [34], [35]. The question whether, and to what extent, patenting of genes impacts academic science has remained a topic of controversy. To follow functional annotation progress of patented versus non-patented genes since 2001, we applied the GCI scoring system to all patented and non-patented genes and followed the fate of the different groups up to September 2007, 6 years after the release of the first draft of the human genome sequence. From NCBI's patented sequence database, we extracted 11278 patented genes, applied the GCI scoring system based on both GeneLynx release 0.9 (May 2001) and Entrez Gene release September 2007 and compared the GCI score distribution for the 2 time points. In the same way, we extracted 20867 non-patented genes from GeneLynx 0.9 and 19331 non-patented genes from Entrez Gene September 2007 and compared the score distributions between the 2 genome releases. It should be noted that there have been fluctuations in the mappings of cDNAs to genes in successive genome releases accounting for the slight variations in the number of genes in the different groups between 2001 and 2007. For patented genes in 2001, 93% of GCI scores spanned across the low to medium part of the scale (GCI<7.5), with merely 7% clustering at the high end (GCI≥7.5) (Figure 5). In 2007, however, the centre of the distribution was shifted from low (GCI = 1.5–2.4) to medium high scores (GCI = 6.5–7.4), with over 36% of genes ranking above 7.4. In 2007, there were only 1.3% of patented genes left scoring below 3.5. By dividing the genes into bins, it became apparent that over 40% of patented genes had climbed up the scale from the low GCI bin, with the highest increase for the high GCI bin (Table 6).

**Table 6:** Change in GCI score distribution of patented versus non-patented genes between May 2001 and September 2007.

GCI bins	Patented	Non-patented
	% (genes*)	% (genes*)
1.0–3.4	−41.3 (−4653)	−15.4 (−4371)
3.5–7.4	+9.7 (+1226)	+12.3 (+2247)
7.5–10.0	+31.6 (+3642)	+3.1 (+588)

* Unbalanced numbers due to gene mapping changes between 2001 and 2007

The evolution for the GCI distribution for non-patented genes was markedly different. As illustrated in Figure 5, in 2001 over 90% of all non-patented genes clustered at scores below 3.5, with less than 1% reaching the high end of the scale (GCI≥7.5). In September 2007, this distribution had not notably changed with over 75% of genes remaining at the low end of the scale (GCI<3.5). The majority of genes that had left the low GCI bin had moved to the medium GCI bin, and not to the high GCI bin as observed for patented genes (Table 6). Also, a fraction of the genes originally in the low score bin had been removed all together (1535 genes), likely due to genome reannotations.

### Accessing the Gene Characterization Index

**Figure 6:**
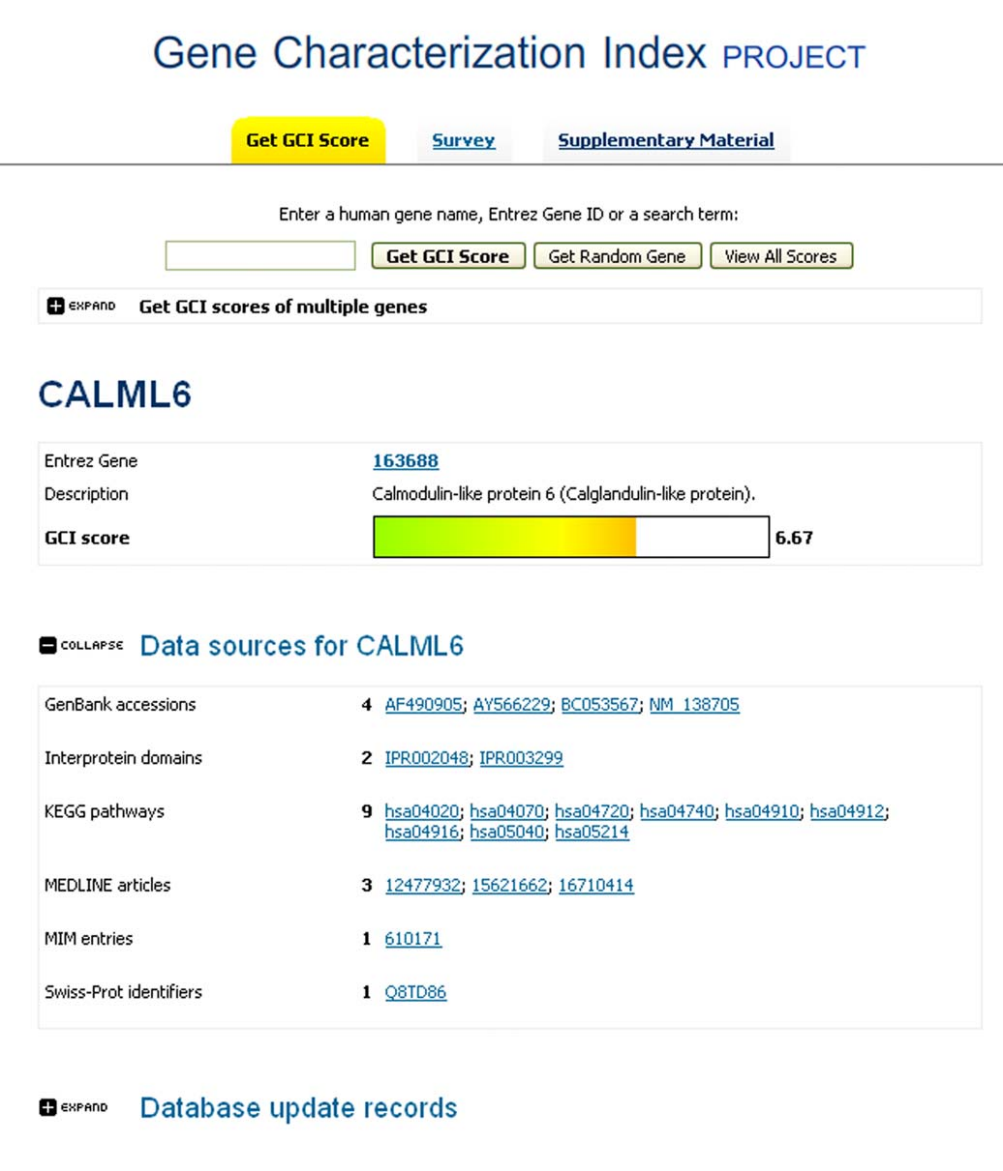
Screenshot of GCI Web Page. Example of Calmodulin-like protein 6 returned by GCI search engine with gene-specific GCI score and links to data sources.

Users may access the GCI at http://www.cisreg.ca/gci/, where they can search for the functional characterization level of their favorite gene, browse for genes with specific annotation levels, choose random genes and further explore the system. After searching with the gene name, Entrez Gene ID or free text, the GCI scoring system returns the GCI score for the searched gene, as well as links to various data sources underlying the computed score (Figure 6). It is also possible to search for genes in bulk, where the GCI scoring system accepts lists of genes with Entrez Gene IDs and returns scores for all genes from the list. For scientists interested in comparing their own perception to the automated scores, they may perform a blinded test. A randomly selected set of 10 genes will be displayed and the user may assign their own scores. After assignments are submitted, a comparison report is generated. Scores submitted through this process will be used as training data for future releases of GCI (to reflect temporal shifts in human perception of what constitutes a well-characterized gene). Finally, users may download the entire set of scores, as well as access the data and Supplementary Material described in this manuscript.

## Discussion

We have introduced a novel bioinformatics procedure to quantitate the functional characterization of each protein-encoding human gene. Based on a reference collection of characterization scores assigned by diverse life scientists, we trained a classification function to predict scores depending on functional annotations in the Entrez Gene database. Using a MARS classifier that performed well in cross-validation, we were able to assign characterization scores to all human genes.

As a first assessment of the performance of the GCI scoring system, we assigned scores to all accessible genes in consecutive releases of the human genome and followed GCI score distribution and attribute selection of the MARS model. We could show that the GCI scoring function mirrored an increase in genome annotation performed by the research community through a shift of score distributions to deeper annotation levels. The changing weight assigned by the GCI scoring system to different attributes over time reflected befittingly the evolution of the human genome. While early releases were marked by predictions of fluctuating numbers of genes, whose active transcription could only be confirmed for a fraction, the association of cDNAs with predicted genes was an important factor in the genome characterization effort. Also, the rate of gene discovery was high, and the classification of novel genes according to sequence motifs and domains into protein families was an important step in inferring functional information. Unlike this early phase of the genome era, the sequence quality of later releases improved dramatically with the official closure of the human genome project in April 2003 [36]. As a consequence, gene numbers became more stable leveling off around 25000 human genes [37]. Most of these genes were confirmed by RNA transcripts and basic functional information existed for many of them. Later advancements in gene annotations captured by the GCI scoring model, therefore, were defined by deeper levels of functional characterization, especially in the form of scientific publications describing individual genes and curated functional knowledge in databases such as KEGG, OMIM and SwissProt.

In a second assessment of model performance, we showed that GCI annotation levels correlated well with Gene Ontology terms of *molecular function*, with no correlations observed for the *biological process* and *cellular component* categories. This observation revealed that GCI scores, which are based on specific gene attributes, referred to unambiguous cellular functions such as binding, receptor, transporter or enzyme activities rather than higher-ranking biological processes or cellular components of a gene's activity. It also became apparent that the level of functional annotation captured by the GCI scoring model increasingly paralleled GO *molecular function* hierarchies the deeper a gene's functional characterization reached.

Applying the GCI to 3 major protein families of current drug targets, we presented each family's overall annotation status and pointed to potential candidates for further investigation within these highly “druggable” gene classes. Most likely due to their great sequence diversity rendering homology searches across species more difficult, the sequencing of the human genome had revealed many new GPCRs. In our survey, this gene class produced the largest fraction of weakly characterized genes leaving ample opportunity for further investigations. The small protein family of nuclear receptors, to the contrary, displayed less room for development with the majority of genes deeply characterized, possibly due to their small number and easy accessibility compared to membrane-bound receptors. Even though both families of ion-channel genes lacked genes remaining at the very low end of the characterization scale, there still was prospect of novel therapeutic applications owing to hitherto untargeted subunits of target protein complexes and family members regulating protein structure. Given that over 50% of current drugs target G protein-coupled receptors, nuclear receptors and ion channels and that the majority of new drugs target precedent domains, to tap the full potential of these families by highlighting any neglect may be important for the advancement of therapeutic approaches by small molecule drugs.

As opposed to the common belief that corporate interest had a negative impact on scientific progress in particular through the filing of patents on genes and gene-derived sequences, we observed that patenting did not hamper gene characterization. As already apparent from the analysis of “druggable” genes both within specific gene classes (Figure 4A) and the whole genome (Figure 4B) and based on the fact that the large majority of drug target genes (86%) was protected by patents, drug targets mostly clustered at the high end of the characterization scale. Analyzing annotation progress of patented versus non-patented genes across an extended time period unveiled an association of functional characterization and gene patenting. While it is probable that patenting was biased towards proteins with detectable protein domains now recorded by genome annotation engines, the increase in the patented gene scores was heavily driven by new publications. The scientific community seemed to have been focusing on patented genes in their efforts to study the molecular function of genes.

Another capacity of the GCI scoring system is to identify areas of “neglect” within either groups of genes or the whole genome. Following the annotation status of all human genes since the release of the first draft of the human genome, we identified a large pool (∼14500) of genes essentially uncharacterized to the present day. These genes have minimal functional annotation with scores below 3.5 and represent over 75% of genes neither protected by patents nor targeted by small molecule drugs. These findings clearly illustrate the inestimable potential still hidden within the human genome in that a considerable portion of genes are shrouded in darkness, awaiting attention and functional elucidation.

Using the GCI scoring system to delineate groups of scarcely annotated genes could aide decision-making for the allocation of research funds. Current research funds are distributed in large part on the basis of extensive preliminary data; there is great reluctance to fund projects deemed as “high-risk” which would include the study of uncharacterized genes. As indicated in this report, vast genome areas have remained unexplored. The availability of GCI scores could direct resources to these neglected areas. During grant reviews, GCI could help identify proposals of greater novelty (a weighting factor for many funding groups) by serving as a novelty measure.

The GCI produced by this study represents a novel direction in bioinformatics research. By classifying genes based on a qualitative score assigned by humans, it represents opinion research. The existing GCI scores reflect the state of opinion at the time of the survey. For instance, in 19 cases evaluators assigned a score of 10 to a gene. While this perceived high characterization reflects the most deeply studied genes, it is unlikely that future scientists would view our present state of gene knowledge as complete. As research advances, the perspectives of scientists will without doubt change and our expectations for the required properties of genes to be considered characterized will become more stringent. In addition, over time, the available types and sources of data change. Therefore, the GCI scoring function will require periodic updating to reflect available annotation resources and changing opinions of researchers. Besides, it would be desirable in the future to develop GCI scores for model organism genes to determine how the characterization of orthologs of human genes influences the progression of the GCI. The GCI scoring system could serve as an important tool for focusing efforts and resources on the study of weakly characterized genes.

## Methods

### Analysis of results from survey

As described, the survey of 52 scientists produced an initial collection of scores for the 100 reference genes with an average of 4.6 scores per gene. We implemented quality control procedures to identify unusual results. For each respondent, we compared the set of assigned gene scores to the mean score for each gene using a Pearson correlation coefficient. Scores from 3 reviewers with correlation scores more than 2 standard deviations from the average correlation score were excluded from the reference collection (Supplementary Figure S2). As an additional quality control procedure, we reviewed individual gene scores for unaccounted deviations from the average. No individual gene or reviewer appeared particularly unusual after this final review, although we identified 3 scores as outside the observed range of scores and thus excluded them. After removal of outlying scores, an average of 4.2 scores per gene remained in the reference set.

### Model selection

We used the R Statistical Language (http://www.r-project.org/) to implement all gene characterization score prediction methods. Initially, we conducted a performance review of linear models (LM), regression trees (RT), neural nets (NN), support vector machines (SVM) and multivariate additive regression splines (MARS). All of the approaches exhibited similar and adequate accuracy for GCI scoring, as assessed by cross-validation.

### Model validation

We verified the quality of the model by a Leave-One-Out (LOO) cross-validation method. For each of the 100 genes, we constructed a model with the other 99 genes and compared the score assigned by the model to the excluded gene to the reference score. The Root Mean Square (RMS) error provided an overall performance measure. We tested a number of data transformations including binary values, binned, log, maximum value normalization, and z-score normalization. Of the 5 model frameworks tested, SVM and MARS performed best. The SVM model failed to assign scores across the entire scale and was therefore set aside. For the MARS model a range of values for the parameters “degree“ (range 1 to 3) and “penalty“ (range 0 to 6) was tested. We presented the best model with degree 1 and penalty of 1. The MARS procedure selected a subset of available attributes to optimize the fit to training data. The “degree” controlled the maximum number of splines that could be used for each attribute across the range of the expected predictions, and the “penalty” was used to decide if an additional attribute should be utilized in constructing the model. Increasing the “penalty” reduced the number of attributes used by the final model. Attributes utilized in the best MARS model are shown in Table 1. A complete documentation of the model validation is provided in the online Supplementary Material.

### Calculation of Resnik scores

The usage of GO terms varies considerably–a rather general GO term may be associated with many genes and is therefore not very explicit, whereas a very detailed term will only be used for few genes for which the specific function may be applied. Resnik self-similarity scores for the GO *molecular function* taxonomy provide a numerical measure for the depth of GO annotations for individual genes. The Resnik self-similarity score is based on the lowest (most detailed) parent node in the GO annotation hierarchy for a specific gene by extracting the maximum information content of the node [22]. The information content is linked to the probability of observing the GO term based on counting how many times the GO term appears in annotated gene products which is reported regularly by the GO Consortium [38]. The GOSim 1.0.2 package [39] was used to compute the scores in the R 2.5.1 statistics package (http://www.r-project.org/).

### Analysis of patented genes

We screened NCBI's patented nucleotide sequence database for human transcripts and matched a total of 14295 GenBank accessions to patent records. To assess GCI score distributions across several years, we mapped the patented genes to either GeneLynx or Entrez Gene identifiers and applied the GCI scoring model based on successive GeneLynx [10] and Entrez Gene [9] releases.

### Gene mappings between GeneLynx and Entrez Gene

For all analyses of datasets comparing data from the latest GeneLynx release (April 2004) and earlier with datasets from more recent Entrez Gene releases, mappings between GeneLynx and Entrez Gene identifiers were performed via EnsEMBL [40] gene and transcript intermediates. Due to substantial changes in gene mappings between May 2001 and September 2007, only 16494 individual genes could be directly mapped between GeneLynx and Entrez Gene with a bias towards genes with more extensive functional annotation (see Supplementary Material).

## Supporting Information

Table S1 Collection of reference genes
(0.08 MB PDF)

Table S2 Initial list of 40 gene attributes
(0.02 MB PDF)

Figure S1 Evaluator-assigned GCI score distribution
(0.02 MB PDF)

Figure S2 Outlier evaluator
(0.01 MB PDF)
