# Supplementary material for: Gene Characterization Index: Assessing the Depth of Gene Annotation
Source: PLoS One. 2008 Jan 23;3(1):e1440. doi: 10.1371/journal.pone.0001440 (PMC2194620; doi:10.1371/journal.pone.0001440)
Supplement: Table S1 — Collection of reference genes (0.08 MB PDF) [file pone.0001440.s001.pdf]

Table S1. Collection of reference genes

Entrez ID	GeneLynx ID	Gene Symbol	Rated GCI	Computed GCI		Description
			June 2003	June 2003	Feb 2006	
4629	111	MYH11	9.0	7.7	7.9	myosin, heavy polypeptide 11, smooth muscle
9019	562	MPZL1	4.0	5.5	5.5	myelin protein zero-like 1
6046	731	BRD2	5.5	7.0	7.4	bromodomain containing 2
1231	769	CCR2	8.67	7.5	10	chemokine (C-C motif) receptor 2
4602	821	MYB	7.0	7.4	7.6	v-myb myeloblastosis viral oncogene homolog (avian)
4635	911	MYL4	8.0	7.9	7.1	myosin, light polypeptide 4, alkali; atrial, embryonic
1310	979	COL19A1	6.0	6.2	6.3	collagen, type XIX, alpha 1
8241	1149	RBM10	5.5	6.5	7.1	RNA binding motif protein 10
6834	1361	SURF1	7.75	6.9	7.1	surfeit 1
9093	1487	DNAJA3	7.0	5.8	7.7	DnaJ (Hsp40) homolog, subfamily A, member 3
1285	1601	COL4A3	8.75	8.5	8.4	collagen, type IV, alpha 3 (Goodpasture antigen)
56649	1723	TMPRSS4	6.33	5.7	5.8	transmembrane protease, serine 4
6789	1750	STK4	7.25	7.1	8.9	serine/threonine kinase 4
6428	2035	SFRS3	6.4	6.8	7.2	splicing factor, arginine/serine-rich 3
5621	2490	PRNP	8.25	9.4	10	prion protein (p27-30) (Creutzfeld-Jakob disease, Gerstmann-Strausler-Scheinker syndrome, fatal familial insomnia)
1857	2491	DVL3	8.5	6.9	8.8	dishevelled, dsh homolog 3 (Drosophila)
8848	2775	TSC22D1	5.0	6.6	7.1	TSC22 domain family, member 1 (transforming growth factor beta-stimulated protein TSC-22)
10367	2813	CBARA1	5.5	4.6	5.0	calcium binding atopy-related autoantigen 1
9976	3119	CLEC2B	6.25	6.0	6.8	C-type (calcium dependent, carbohydrate-recognition domain) lectin, superfamily member 2 (activation-induced)
7273	3244	TTN	8.5	8.5	9.6	titin
8187	3349	ZNF239	4.83	5.3	5.6	zinc finger protein 239
388	3451	RHOB	7.2	6.7	7.7	ras homolog gene family, member B
8498	3656	RANBP3	7.25	5.0	6.3	RAN binding protein 3
2692	3863	GHRHR	8.0	7.3	9.6	growth hormone releasing hormone receptor
9509	3944	ADAMTS2	7.8	6.6	6.7	a disintegrin-like and metalloprotease (reprolysin

						type) with thrombospondin type 1 motif, 2
3613	4115	IMPA2	6.5	6.8	7.9	inositol(myo)-1(or 4)-monophosphatase 2
5581	4138	PRKCE	8.0	8.9	10	protein kinase C, epsilon
3212	4497	HOXB2	7.67	7.1	7.5	homeo box B2
7569	4631	ZNF182	4.67	5.1	5.1	zinc finger protein 182 (ZNF21; zinc finger protein 21) (KOX 14)
4049	4677	LTA	7.8	7.7	9.5	lymphotoxin alpha (TNF superfamily, member 1)
7534	4897	YWHAZ	9.25	7.6	9.2	tyrosine 3-monooxygenase/tryptophan 5-monooxygenase activation protein, zeta polypeptide
332	24855	BIRC5	7.29	8.0	8.0	baculoviral IAP repeat-containing 5 (survivin)
3875	4950	KRT18	8.33	7.7	9.7	keratin 18
6609	5535	SMPD1	8.25	8.3	9.8	sphingomyelin phosphodiesterase 1, acid lysosomal (acid sphingomyelinase)
5371	5615	PML	8.0	8.0	8.0	promyelocytic leukemia
2649	5624	NR6A1	5.6	7.3	7.7	nuclear receptor subfamily 6, group A, member 1
3709	5694	ITPR2	6.8	6.7	6.4	inositol 1,4,5-triphosphate receptor, type 2
4036	6134	LRP2	9.0	7.6	9.7	low density lipoprotein-related protein 2
222	6201	ALDH3B2	8.0	6.8	7.3	aldehyde dehydrogenase 3 family, member B2
861	18185	RUNX1	7.25	8.0	9.3	runt-related transcription factor 1 (acute myeloid leukemia 1; aml1 oncogene)
10782	6289	ZNF274	5.67	4.9	6.2	zinc finger protein 274
24138	6576	IFIT5	5.25	4.1	4.5	interferon-induced protein with tetratricopeptide repeats 5
2494	6623	NR5A2	5.75	7.3	9.2	nuclear receptor subfamily 5, group A, member 2
9552	7404	SPAG7	4.33	4.0	5.5	sperm associated antigen 7
81	7546	ACTN4	8.6	7.8	9.5	actinin, alpha 4
683	7690	BST1	7.0	7.7	7.9	bone marrow stromal cell antigen 1
8644	7886	AKR1C3	5.5	6.8	8.6	aldo-keto reductase family 1, member C3 (3-alpha hydroxysteroid dehydrogenase, type II)
3014	7932	H2AFX	8.0	7.1	7.6	H2A histone family, member X
23054	7977	NCOA6	6.2	6.0	7.2	nuclear receptor coactivator 6
8832	8146	CD84	4.75	5.9	5.8	CD84 antigen (leukocyte antigen)
6863	8203	TAC1	7.75	7.5	8.9	tachykinin, precursor 1 (substance K, substance P,

						neurokinin 1, neurokinin 2, neuromedin L, neurokinin alpha, neuropeptide K, neuropeptide gamma)
23450	8411	SF3B3	8.0	6.5	7.1	splicing factor 3b, subunit 3, 130kD
3884	8466	KRTHA3B	7.25	5.6	7.0	keratin, hair, acidic, 3B
4312	8517	MMP1	8.5	7.9	8.8	matrix metalloproteinase 1 (interstitial collagenase)
79192	8533	IRX1	4.25	5.0	5.5	iroquois homeobox protein 1
887	8745	CCKBR	8.6	7.3	9.3	cholecystokinin B receptor
5781	9462	PTPN11	7.75	8.3	10	protein tyrosine phosphatase, non-receptor type 11 (Noonan syndrome 1)
5311	24923	PKD2	7.0	9.6	8.1	polycystic kidney disease 2 (autosomal dominant)
26118	9762	WSB1	2.5	4.6	6.1	SOCS box-containing WD protein SWiP-1
9478	9789	CABP1	6.0	5.9	7.0	calcium binding protein 1 (calbrain)
55635	10729	DEPDC1	2.25	3.5	4.0	DEP domain containing 1 (SDP35) (formerly hypothetical protein FLJ20354)
27327	4059	TNRC6A	3.0	2.0	6.1	trinucleotide repeat containing 6A (formerly known as KIAA1460 protein)
55656	10796	INTS8	2.33	3.1	4.3	integrator complex subunit 8 (formerly hypothetical protein FLJ20530)
85329	10816	LGALS12	5.5	5.7	5.5	lectin, galactoside-binding, soluble, 12 (galectin 12)
7462	10854	LAT2	5.5	5.5	6.0	linker for activation of T cells family, member 2 (WBSCR5, Williams-Beuren syndrome chromosome region 5)
23413	10862	FREQ	7.2	7.1	7.3	frequenin homolog (Drosophila)
23786	11124	BCL2L13	4.8	4.6	6.0	BCL2-like 13 (apoptosis facilitator)
57154	11134	SMURF1	7.0	7.0	9.0	E3 ubiquitin ligase SMURF1
26017	11147	FAM32A	1.75	3.7	3.9	family with sequence similarity 32, member A (formerly DKFZP586O0120 protein)
51227	11323	PIGP	6.75	6.0	7.6	phosphatidylinositol glycan, class P (Down syndrome critical region gene 5)
4289	11526	MKLN1	4.5	4.3	6.1	muskelin 1, intracellular mediator containing kelch motifs
51300	11559	C3orf1	4.0	4.0	4.5	chromosome 3 open reading frame 1
Discontinued	12193	PRO1410	1.25	1.7	NA	Homo sapiens PRO1410 mRNA, complete cds
56910	12892	STARD7	3.75	3.4	5.2	START domain containing 7
54897	13179	CASZ1	2.0	2.8	5.0	castor homolog 1, zinc finger

						(Drosophila) (formerly hypothetical protein FLJ20321)
Discontinued	13375	AK000769	1.5	1.5	NA	Homo sapiens cDNA FLJ20762 fis, clone HEP00177
22950	13677	SLC4A1AP	4.0	4.6	5.7	solute carrier family 4 (anion exchanger), member 1, adaptor protein
221061	14104	C10orf38	1.75	1.7	3.7	chromosome 10 open reading frame 38
118987	14474	PDZD8	2.5	1.8	5.3	PDZ domain containing 8
2688	15457	GH1	7.4	8.2	9.2	growth hormone 1
1915	15739	EEF1A1	7.33	7.9	7.9	eukaryotic translation elongation factor 1 alpha 1
56269	16593	IRGC	2.75	2.3	2.9	immunity-related GTPase family, cinema (formarly hypothetical protein R30953_1)
80142	18355	PTGES2	4.6	4.8	8.1	prostaglandin E synthase 2
9907	18824	KIAA0415	1.4	1.5	3.9	KIAA0415 protein
57722	19965	NOPE	4.0	4.0	4.2	likely ortholog of mouse neighbor of Punc E11
283537	20480	LOC283537	1.5	1.5	4.1	hypothetical protein LOC283537
84331	21232	C16orf14	1.75	2.8	3.7	chromosome 16 open reading frame 14
NA	21396	AF339787	1.67	1.5	NA	Homo sapiens clone IMAGE:205688, mRNA sequence
23594	15275	ORC6L	6.25	5.4	8.2	origin recognition complex, subunit 6 homolog-like (yeast)
984	24653	CDC2L1	6.33	8.6	8.7	cell division cycle 2-like 1 (PITSLRE proteins)
NA	119957	-	1.67	1.5	NA	ESTs
NA	120742	-	1.0	1.5	NA	ESTs
NA	121617	-	1.25	1.5	NA	ESTs, Weakly similar to A43932 mucin 2 precursor, intestinal [H.sapiens]
NA	123306	-	1.2	1.5	NA	ESTs
NA	123342	-	1.2	1.5	NA	ESTs
NA	124248	-	1.75	1.5	NA	ESTs
NA	127008	-	1.25	1.5	NA	ESTs, Moderately similar to ALU7_HUMAN ALU SUBFAMILY SQ SEQUENCE CONTAMINATION WARNING ENTRY [H.sapiens]
NA	127744	-	1.5	1.5	NA	ESTs
NA	130377	-	1.0	1.5	NA	ESTs
NA	130666	-	1.0	1.5	NA	ESTs
4629	111	MYH11	9.0	7.7	7.9	myosin, heavy polypeptide 11, smooth muscle
