# Supplementary material for: Gene Characterization Index: Assessing the Depth of Gene Annotation
Source: PLoS One. 2008 Jan 23;3(1):e1440. doi: 10.1371/journal.pone.0001440 (PMC2194620; doi:10.1371/journal.pone.0001440)
Supplement: Table S2 — Initial list of 40 gene attributes (0.02 MB PDF) [file pone.0001440.s002.pdf]

Table S2. Initial list of 40 gene attributes

Attribute	Description
BLOCKS	Links to Blocks database
DBSNP	links to Single Nucleotide Polymorphism database
DESCRIPTION	Functional gene description
EGAD	Links to Expressed Gene Anatomy database
ENSEMBL_GENOMIC	Genomic sequence information in Ensembl
ENSEMBL_PROTEIN	Protein sequence information in Ensembl
ENSEMBL_TRANSCRIPT	Transcript sequence information in Ensembl
EST	Expressed Sequence Tag
GBACC	GenBank sequences
GDB	Links to Human Genome Database
GENECARDS	Links to GeneCards database
GENOMIC	Genomic sequences
GENPEPT	Links to GenPept protein database
GO	Gene Ontology annotations
HGBASE	Links to Human Genic Bi-Allelic Sequences database
HOMOLOGENE	Non-human homologous sequences (NCBI)
HSSP	Links to database of homology-derived secondary structure of proteins
HUGO	HUGO gene symbol
HUMAN_MOUSE	Human mouse conserved sequences
INTERPRO	Interpro domains
KEGG	KEGG pathways
LOCUSLINK	LocusLink (NCBI)
MEROPS	Links to Merops peptidase database
OMIM	Online Mendelian Inheritance In Man (NCBI)
MIPS	Munich information center for protein sequences
MOUSE_HUMAN_MAP	Human mouse comparative map
NAME	Gene symbols and alias symbols
NOMENCLATURE	Standard gene nomenclature
PDB	Links to Protein Data Bank
PFAM	Links to Pfam database
PIR	Protein Information Resource
PRINTS	Links to Prints (protein fingerprints) database
PROSITE	Links to database of protein domains, families and functional sites
PROTEOME	Links to HumanPSD database
REFSEQ	Reference sequences (NCBI)
RZPD	German Resource Center for Genome Research
SPID	SwissProt protein links
TREMBL	Links to Trembl database
UNIGENE	UniGene transcriptome (NCBI)
MEDLINE	MEDLINE references in Entrez Gene
