# Supplementary figures and images for: Gene Characterization Index: Assessing the Depth of Gene Annotation
Source: PLoS One. 2008 Jan 23;3(1):e1440. doi: 10.1371/journal.pone.0001440 (PMC2194620; doi:10.1371/journal.pone.0001440)

**Figure S1.** Evaluator-assigned GCI score distribution

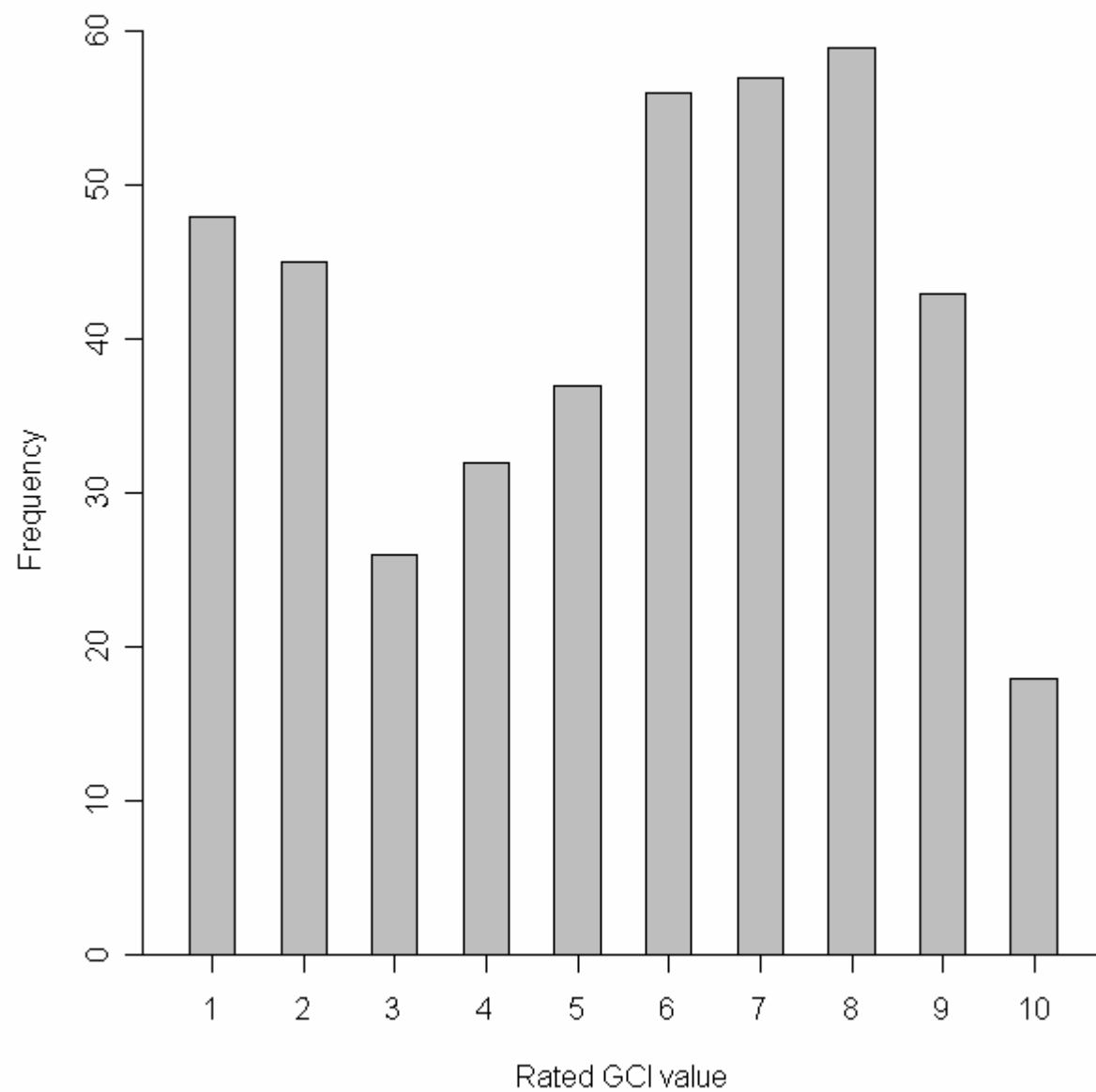

Supplement: Figure S1 — Evaluator-assigned GCI score distribution (0.02 MB PDF) [file pone.0001440.s003.pdf]

**Figure S2.** Outlier evaluator

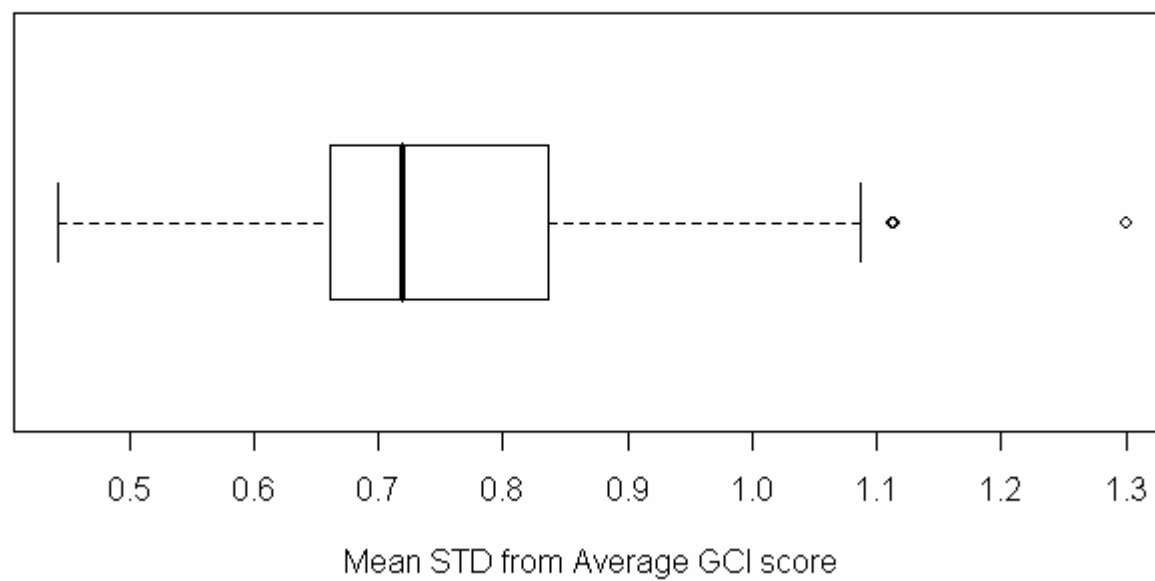

Supplement: Figure S2 — Outlier evaluator (0.01 MB PDF) [file pone.0001440.s004.pdf]
